# What Is the Magnitude and Long-term Economic Cost of Care of the British Military Afghanistan Amputee Cohort?

**DOI:** 10.1007/s11999-015-4250-9

**Published:** 2015-06-01

**Authors:** D. S. Edwards, Rhodri D. Phillip, Nick Bosanquet, Anthony M. J. Bull, Jon C. Clasper

**Affiliations:** 1 Royal Centre for Defence Medicine, Birmingham, UK; 2 The Royal British Legion Centre for Blast Injury Studies, Imperial College, London, SW7 2AZ UK; 3 Defence Medical and Rehabilitation Centre, Headley Court, UK; 4 Department of Bioengineering, Imperial College, London, UK

## Abstract

**Background:**

Personal protection equipment, improved early medical care, and rapid extraction of the casualty have resulted in more injured service members who served in Afghanistan surviving after severe military trauma. Many of those who survive the initial trauma are faced with complex wounds such as multiple amputations. Although costs of care can be high, they have not been well quantified before. This is required to budget for the needs of the injured beyond their service in the armed forces.

**Question/purposes:**

The purposes of this study were (1) to quantify and describe the extent and nature of traumatic amputations of British service personnel from Afghanistan; and (2) to calculate an estimate of the projected long-term cost of this cohort.

**Methods:**

A four-stage methodology was used: (1) systematic literature search of previous studies of amputee care cost; (2) retrospective analysis of the UK Joint Theatre Trauma and prosthetic database; (3) Markov economic algorithm for healthcare cost and sensitivity analysis of results; and (4) statistical cost comparison between our cohort and the identified literature.

**Results:**

From 2003 to 2014, 265 casualties sustained 416 amputations. The average number of limbs lost per casualty was 1.6. The most common type of amputation was a transfemoral amputation (153 patients); the next most common amputation type was unilateral transtibial (143 patients). Using a Markov model of healthcare economics, it is estimated that the total 40-year cost of the UK Afghanistan lower limb amputee cohort is £288 million (USD 444 million); this figure estimates cost of trauma care, rehabilitation, and prosthetic costs. A sensitivity analysis on our model demonstrated a potential ± 6.19% variation in costs.

**Conclusions:**

The conflict in Afghanistan resulted in high numbers of complex injuries. Our findings suggest that a long-term facility to budget for veterans’ health care is necessary.

**Clinical Relevance:**

Estimates here should be taken as the start of a challenge to develop sustained rehabilitation and recovery funding and provision.

## Introduction

The year 2014 saw the wind down and withdrawal of British troops from Afghanistan; over the past 2 years, military operations have progressively been handed over to the Afghan security forces. It is now well documented that the conflict is associated with casualties surviving highly complex military-related trauma. This has been attributed to improved personal protection equipment, improved on-the-ground medical care, and rapid extraction of the casualty [3, 7]. However, one consequence of the complexity of the wounds is illustrated by the resulting multiple amputees [2].

Previous studies have provided snapshots of the magnitude of the amputee figures from military operations in Afghanistan but with the draw down, only now is the complete size of the amputee cohort evident [8, 10]. Medical care and research is now focused on the long-term care of our injured service personnel as well as capturing the lessons learned from Afghanistan and through research and training ensuring that this is not lost. With evidence from Vietnam [11] suggesting that medical and rehabilitative needs extend beyond 25 years after injury, we have an imperative to assess the magnitude of the amputee cohort and the long-term health economics for provision of care for contemporary casualties. An accurate description of the casualty statistics is necessary to calculate healthcare spending and long-term care costs to aid the healthcare policy decision-making process.

The purpose of this study is twofold. First it is necessary to quantify and qualify the extent and nature of all trauma-related amputations from Afghanistan. This will provide us with a compete profile of the amputee cohort detailing the exact injuries sustained. Second, using these data, and after examining published work on long-term care of amputees and mathematical algorithms of health economics, we aim to estimate the projected long-term cost of the Afghanistan amputation cohort from the perspective of the healthcare provider, the National Health Service.

## Materials and Methods

**Fig. 1:**
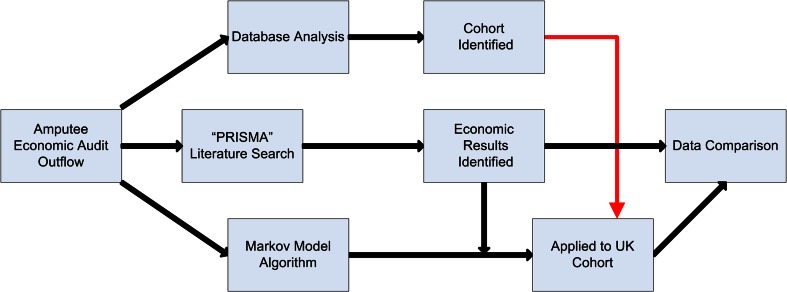
A flowchart demonstrating the study process used during the research.

The study was conducted in a stepwise manner (Fig. 1).

Patients with trauma-related amputations were identified from the definitive database, based at Headley Court, used for prosthetic fitting, and the Joint Theatre Trauma database based at the Royal Centre for Defence Medicine. Data extracted included number of amputations, locations and level of amputations, and date of injury. After testing for normality, statistical analysis was performed using Student’s t-test and Mann-Whitney U test and chi-square test where appropriate using SPSS statistics Version 20.1 (SPSS Inc, Chicago, IL, USA) with significance set at p < 0.05.

A Preferred Reporting Items for Systematic Reviews and Meta-Analyses (PRISMA) type search was performed using the OvidSP platform using two separate search resources (PubMed/MEDLINE and Embase) to identify research articles that calculate and explain in economic terms the cost of rehabilitation of amputees [19]. Articles in both the civilian and military setting were examined. This database was searched for references using the search terms “Amputee”, “rehabilitation”, and “cost” as keywords. Primary and secondary exclusion criteria were then used to filter the results. Primary exclusions included audiovisual, lecture, book, and biography publications. Secondary exclusions included letters, retracted articles, comments, editorials, and conference papers. Manual strategies subsequently used to establish relevance were (1) title review; (2) Medical Subject Headings [17] term review; (3) abstract review; and finally (4) full article review. Papers detailing cost analyses of the long-term cost of amputee rehabilitation were evaluated and a comparison of their findings made providing the authors revealed specific costs of prosthetic care and examined or performed a sensitivity analysis, long-term costs, and tolerances in their economic findings.

**Fig. 2A–B:**
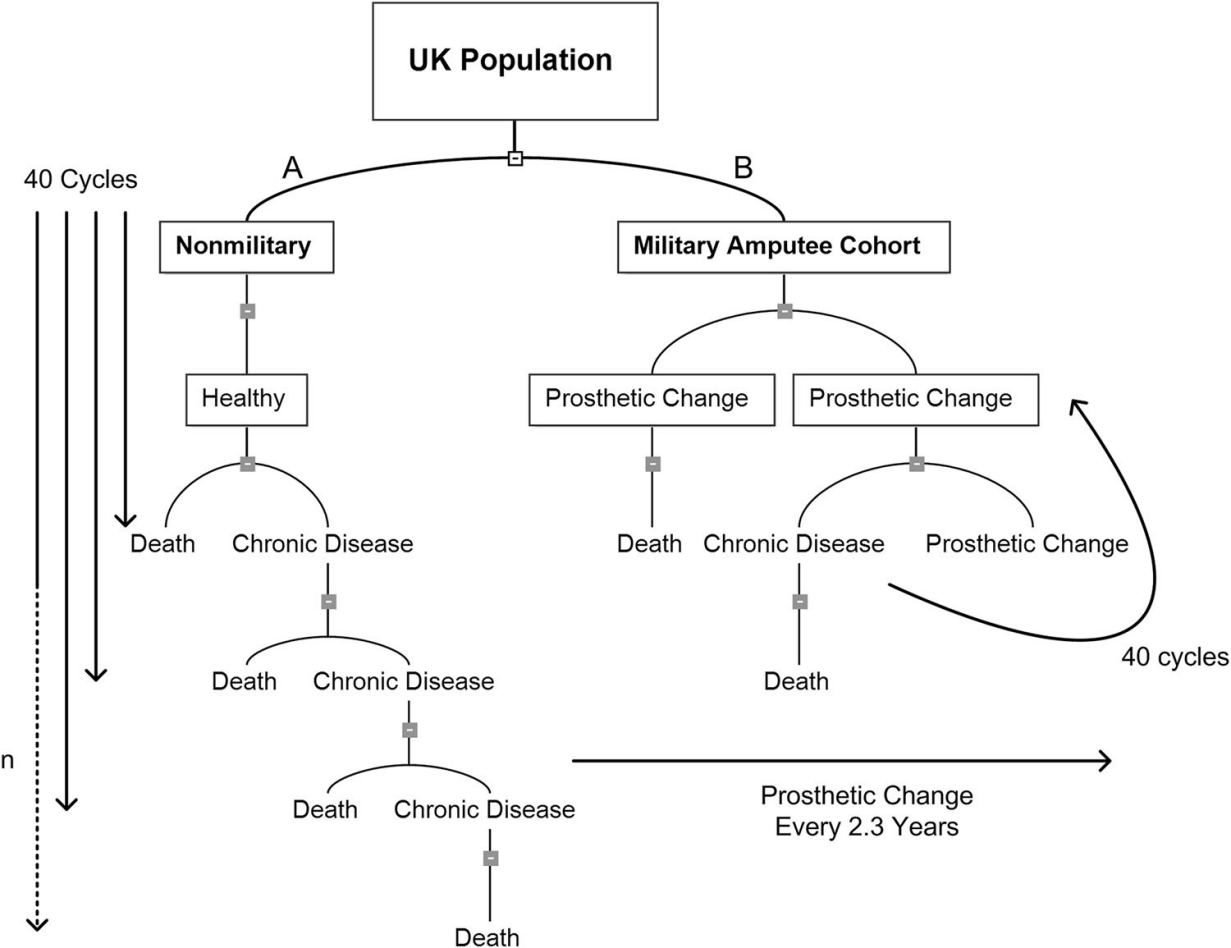
A Markov decision tree demonstrating the difference in the chronic health profile of (**A**) the normal population and (**B**) the military amputee cohort described in this work.

The Markov model of economic evaluation of health uses both resource management and health outcome consequences from medical intervention. Markov models represent a stochastic process used in the economic evaluation of health and disease [5]. They are used by health economists in the calculation of cost of health care and intervention, particularly in chronic disease. In this model the economics relating to death from disease, disease progression, effectiveness of treatment, and natural death risk are applied to annual cycles of care as the patient ages. An adapted Markov model algorithm was used to calculate the cost of rehabilitation and prosthetic costs on an assumed average patient in our amputee cohort in the British market. In our Markov decision tree, the decision to treat arm is replaced with actual treatment in the form of fitting a new prosthetic device. Prosthetics costs were cycled every 2.3 years and run in parallel with 1-year Markov cycles (Fig. 2). Only direct costs relating to prosthetics, consumables and related clinical activities were considered. Model assumptions such as time horizon (40 years), prosthetic costs, and prosthetic cycle length (2.3 years) were derived from our literature review of amputee health economics [4, 9, 16]. Where probabilistic assumptions necessary for the model (such as probability of wound complications, likely timing of chronic diseases, frequency of clinic visits, projected prosthetic advancements, and subsequent additional replacements) were not available, coauthors were consulted whose specialist fields include orthopaedic surgeons (DSE, JCC), a rehabilitation physician (RDP), an economist (NB), and a bioengineering expert (AMJB). The cost increase per year per casualty was calculated and extrapolated every year for 5 years, then at 5-year intervals until 40 years after the injurious event. A calculation of total cost of the entire cohort was then made using historical economic data from the Bank of England [1]; the costs among studies were normalized for exchange rate and inflation variable. The results from the cost analysis were then compared with the financial findings from the literature search previously performed. Statistical analysis was performed using Pearson’s correlation coefficient to examine the extent of a linear relationship between the literature and this work using SPSS statistics Version 20.1 with significance set at p < 0.05.

Finally, a sensitivity analysis was performed in an attempt to present the potential extremes of valuations and therefore the errors in any assumptions made. Varying prosthetic use and an equal probability of each transition state (health, chronic health, death) of 0.33 was added to our model [4–6]. TreeAge Pro Healthcare 2015 software (TreeAge Software Inc, Williamstown, MA, USA) was used in our model-making and simulations.

## Results

### Extent and Nature of Amputations From British Operations in Afghanistan

From 2003 to October 2014, 265 casualties sustained a total of 416 amputations either at the point of wounding or subsequent to wounds (Table 1). As a result of small casualty figures on the triple and quadruple amputee group, data have been compressed to “Triple +” to prevent the likelihood of an individual being identified and compromising his or her right to medical confidentiality.

**Table 1:** Total amputations per year and type

Year	Single	Double	Triple+	Total	Total number of limbs
2006	^#^	^#^	^#^		
2007	9	^#^	^#^		
2008	19	7	^#^		
2009	20	17	^#^		
2010	34	32	9		
2011	30	20	7		
2012	15	20	^#^		
2013	7	^#^	^#^		
2014	^#^	^#^	^#^		
Total	140	101	24	265	416

Data presented as “#” has been suppressed in accordance with Defence Statistics rounding policy and medical confidentiality.

The mean number of limbs lost per casualty was 1.6 (SD ± 0.68) with a mean range of one in 2006 to 1.7 in 2010. The most common injury pattern per casualty seen was that of a single amputation. When limbs lost per injury type were analyzed, double amputations were the most common. The crossover between single and double amputations occurred at the end of 2008.

The most common level of amputation was transfemoral totaling 153 patients followed by 143 transtibial amputations with peak incidence of both seen in 2010. Single amputations were more likely to be associated with transtibial-level injuries (89 patients out of 140 single amputations), whereas transfemoral amputations were most common in double and triple amputees (134 amputations out of 268 limbs lost). Other levels seen were hind or forequarter, through-knee or knee disarticulation, and foot amputations. When further analyzing double amputees, the most common patterns of injury seen were that of a transfemoral-transfemoral casualty (38 patients out of a total 101 double amputees). Numbers were not sufficient to test this statistically.

### Long-term Costs of Amputee Care

We calculate the long-term cost of the UK Afghanistan lower limb amputee cohort only to be £288 million (USD 444 million) in today’s currency.

**Fig. 3:**
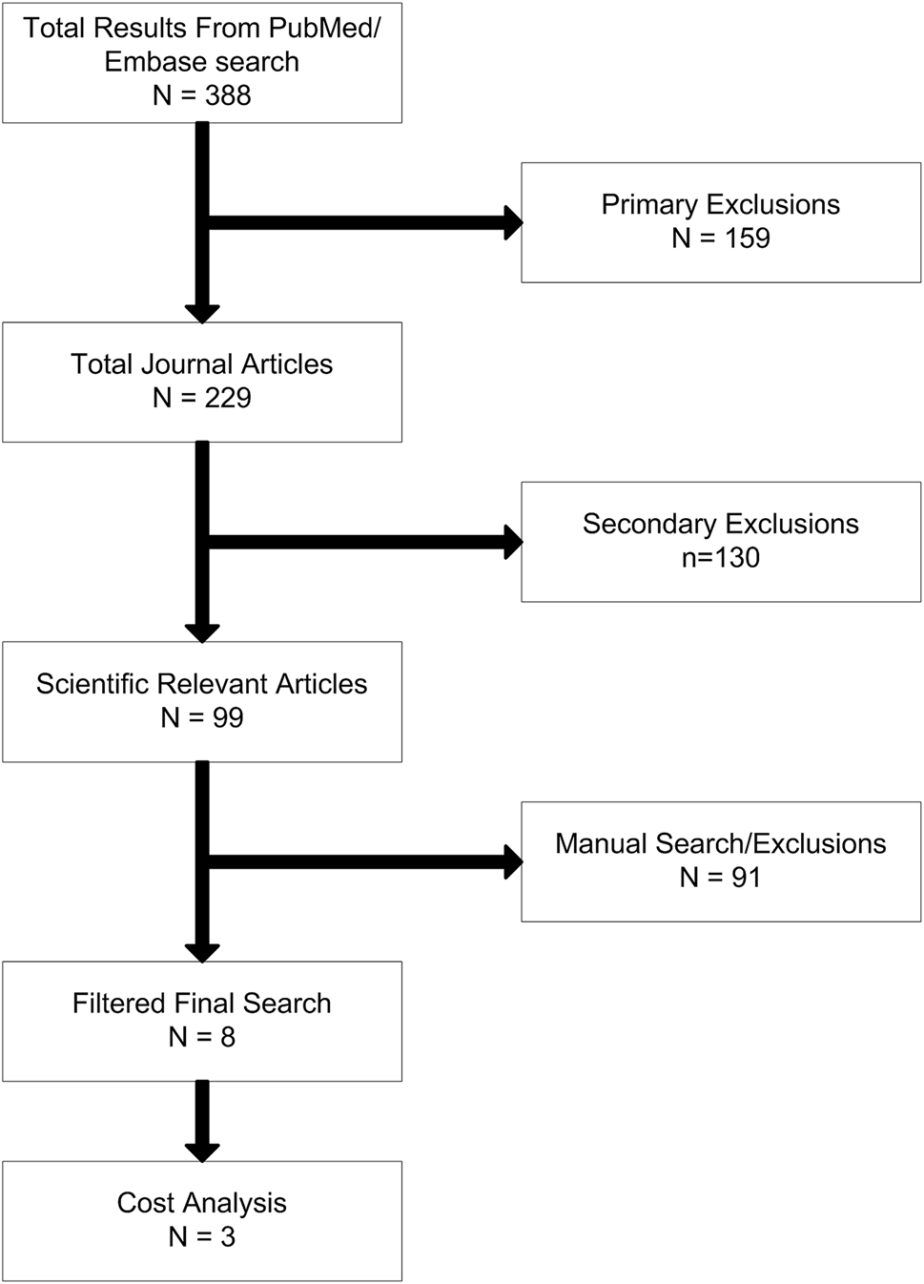
The flowchart systematic review process was used to filter articles found during the literature search.

After removal of duplications from the literature search, we have referred to three papers (Blough et al. [4], Chung et al. [9], and Hertel et al. [16]) to fully detail the entire expected financial implication of trauma-related amputations (Table 2; Fig. 3).

**Table 2:** A summary of papers relevant to actual cost of amputee care

Study	Year	Patient group	Time scale	Comparative study
Hertel et al. [16]	1996	Civilian trauma	2–4 years	Amputation versus reconstruction
Chung et al. [9]	2009	Civilian trauma	2 years to lifetime	Amputation versus salvage
Blough et al. [4]	2010	Military trauma (OIF/OEF)	5, 10, 20 years and lifetime	Vietnam versus OIF/OEF

OIF = Operation Iraqi Freedom; OEF = Operation Enduring Freedom.

Blough et al. [4] estimated the prosthetic cost and care for service men and women with major limb loss specifically related to veterans of Iraq and Afghanistan, Operation Iraqi Freedom (OIF), and Operation Enduring Freedom (OEF). They assessed unilateral upper limb, unilateral lower limb, bilateral upper limb, and multiple limbs. An assembled team of experts processed data collected from a total of 283 veterans using MediCare 2005 prosthetic costing and Markov model analysis. They calculated that in 2010 the 5-year and lifetime cost range for each group was USD 0.11 million and USD 0.82 million (unilateral upper limb), USD 0.23 million and USD 1.46 million (unilateral lower limb), USD 0.33 million and USD 2.12 million (bilateral upper limb), and USD 0.45 million and USD 2.90 million (multiple limbs).

Within the civilian trauma setting, both Hertel et al. [16] and Chung et al. [9] sought to formulate the cost of amputation care. Both papers compare amputations with reconstruction/salvage in severe fractures of the lower limb. Hertel et al.’s [16] paper from 1996 comments that the second and fourth year mean annual cost for the care of the amputee was 24,824 Swiss Francs (SF) and 15,112 SF, respectively. Using Bank of England archives of historic exchange rates and the Bank of England Inflation Calculator© [1], this amounts to annual cost in 2012 of £104,609 (USD 161,350) and £63,679 (USD 98,219), respectively.

Chung et al.’s [9] data were in a contemporaneous cohort of patients and data extracted from the Lower Extremity Assessment Project (LEAP), a multicenter prospective outcome study examining severe limb-threatening injuries, detailed prosthetic, and nonprosthetic costs. They described the cost to the taxpayer in 2009 for the care of amputees to significantly reduce after 2 years, from a total of approximately USD 91,000 for the first 2 years followed by annual ongoing costs of USD 3700 thereafter for life with an additional USD 10,200 every 2 years for additional prosthetics. This equates to a total of £27,000 (USD 45,500) annually for the first 2 years and a total of £69,000 (USD 117,400) for the first 5 years of care. We compared costs as estimated by the three papers we included based on our systematic review [4, 9, 16] and found no differences between them (p = 0.19).

**Fig. 4:**
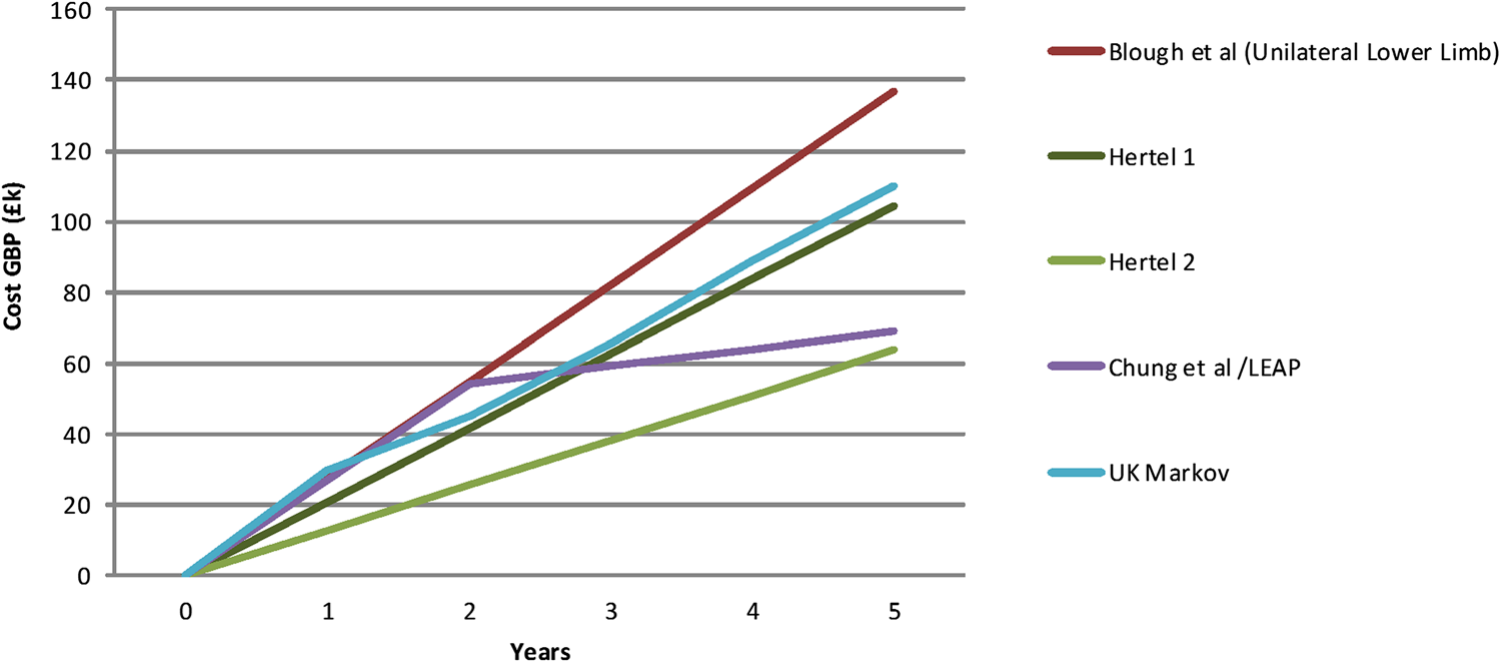
A graph of the projected 5-year costs of amputee healthcare demonstrating the data published in the literature compared with the simplified UK cohort Markov model constructed during our research.

Following Markov model calculations, no statistical differences could be found between our results and published figures in the first 5 years of care, and as a consequence, costing can be considered comparative (Fig. 4).

**Fig. 5:**
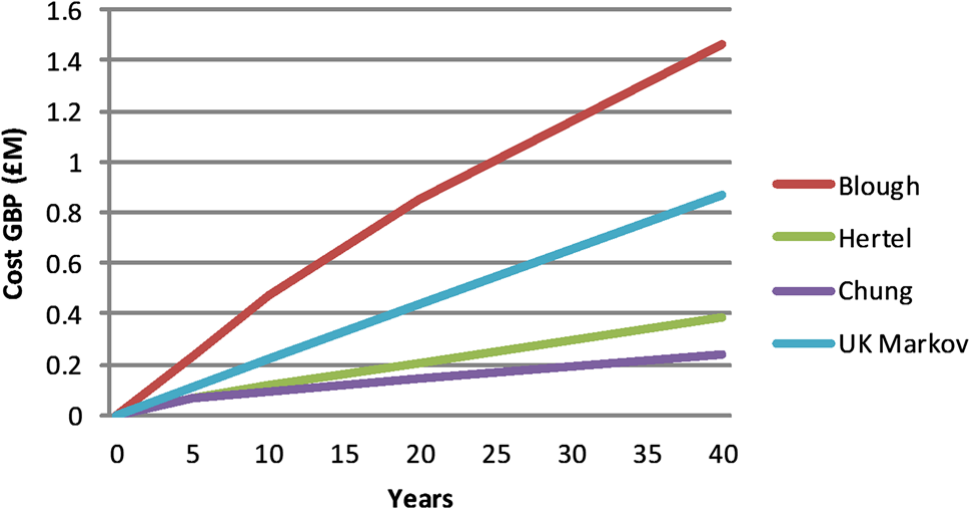
A graph of the projected 40-year costs of amputee health care demonstrating the data published in the literature compared with the simplified UK cohort Markov model constructed during our research.

Blough et al. [4] calculated lifetime cost. Their model suggested that 40 cycles, or years, after the initial injury was an adequate mean point for life expectancy of the cohort taking into account chronic health of a normal population. The healthcare costs of amputees calculated in the searched papers were also extrapolated to the 40-year point described by Blough et al. (Fig. 5). Final discrepancy in the model here and other figures may reflect the additional costs of chronic disease of age, which are factored in the Markov model. Although a major difference in the endpoint figures are seen between Blough et al.’s [4] data and Chung et al.’s [9] work, no difference exists between the data presented here and other figures.

Using data collected in the first part of the study and estimated costs from the economic model findings, entire lower limb amputee cohort long-term costs were subsequently calculated. Based on limb prosthetic costs from Blough et al.’s [4] paper, a single tibial prosthetic cost was £9946 (USD 16,690), a knee prosthesis was £27,154 (USD 56,563), a femoral prosthesis was £27,154 (USD 45,563), and a foot prosthesis was £9747 (USD 14,187). Therefore, the base cost over 40 years of single amputees was £0.87 million (USD 1.34 million) for a transtibial amputee, £1.16 million (USD 1.79 million) for a through-knee amputee, and £1.16 million (USD 1.79 million) for a transfemoral amputee. Additional limb 40-year costs, as seen in double amputations, were calculated as £0.16 million (USD 0.25 million) for a tibial component, £0.45 million (USD 0.69 million) for a through-knee prosthesis, and £0.45 million (USD 0.69 million) for a femoral prosthesis. Therefore, total 40-year costs for double and triple amputees (upper limb not taken into account) are £1.03 million (USD 1.59 million) for a double transtibial, £1.32 million (USD 2.04 million) for transtibial-through knee/transfemoral, and £1.60 million (USD 2.47 million) for a double through-knee/transfemoral.

The sensitivity analysis on our decision-making tree, if “worst case” scenario of equal transition probability is assumed, a variation of ± 6.19% of our total figure is possible if prosthetic change requirements were adjusted from every 2 years to 5 years form the calculated 2.3 years. This resulted in a variation of +2.99% to −10.75% in the overall costs in prosthetics alone.

## Discussion

The hallmark of the wounded legacy from Afghanistan is that of the traumatic amputee. This is likely to be the result of the fact that the improvised explosive device has become the weapon of choice against coalition forces by insurgent groups. With an increase in casualty numbers from this type of weapon as a result of improved casualty evacuation, medical treatment, and personal equipment, large cohorts of severely injured service personnel are returning home requiring multidisciplinary care in the short and long term. In this article, our aim was to formalize our casualty figures that fall into this group and attempt to calculate the cost of their care for future policymaking decisions.

Specific limitations include the small number of suitable publications encountered in our systematic review to guide the analysis. This is the result of the fact that in the civilian setting, trauma-related amputations remain relatively uncommon with peripheral vascular disease and diabetic complications the leading cause of amputation in the developed world. As a consequence, only two papers pertaining to the cost of care of civilian amputees could be used in our analysis [9, 16]. In addition to this, uncertainty on the actual cost of care will undoubtedly result in variation of our calculations. Some of our figures used were costs calculated in the United States, where a larger market drives prices. It is recognized that a 29% difference in price is seen between private and state-funded care [15]. Equally, consumable discounted rates, the effect of inflation, and exchange rate all play a part in producing an error in readings [12]. An assumption that all lower limb amputees would use prosthetics was made. This, however, as demonstrated by Dougherty et al. [11], is not always the case. Costs incurred by a wheelchair user are not negligible; wheelchair types (transit, electric, narrow, prescriptive), house modification, annual servicing, replacement, and caregiver for self-propelled wheelchairs all contribute to costs of care but were not considered in our analysis. Another confounding factor leading to variations in cost is the chronic disease profile of our veterans. It is possible and likely, but currently unknown, that the increased complexity of injuries seen in our cohort will result in different long-term health profiles, including death rate, than that of the general population that the Markov model used relied on. When compared with published data about civilian populations, gender, age, and physical activity in military populations will also demonstrate differing pathological profiles with time. The rehabilitation of amputees from a veteran cohort does not remain within the remit of musculoskeletal medicine and represents the “tip of the iceberg” concept where primary injury results in chronic health issues [13, 14]. Polytrauma, traumatic brain injury, and posttraumatic stress disorder are discussed as the primary medical issues, whereas limited mobility, weight gain, diabetes mellitus, coronary artery disease, chronic obstructive pulmonary disease, and liver failure are secondary/tertiary sequelae of the primary insult. The timeframe of disease progression was to 2035. The subsequent chronic health issues are not considered by the Blough group [4].

The Markov model as set out works on variables and policies on a no-change basis and these generate a certain level of costs. The costs generated are comparable with those in US studies as well as with those in the only previous UK study. However, we should stress that the costs do not cover the costs of any comorbidity or treatment, which may be required for other illness in the future nor do they cover the economic losses that would result if patients have to drop out of the workforce. The altered chronic health profile is described and hypothesized by Geiling et al. [14]. Technological advances in the expanding field of prosthetic use will also have an impact on the cost of care. It would be realistic to assume that including additional treatment cost and economic losses, the total cost or “disease burden” would be much higher than £288 million (USD 444 million) over 40 years; for example, upper limb provision was not included as a result of multiple unknown factors (future costs, future technological advancements, long-term use) and, as a consequence, an uplift in final expenditure would also be expected if included. Our estimates here should be taken as the start of a challenge to develop sustained rehabilitation and recovery funding and provision. To fully appreciate the long-term health consequences of the modern-day military blast victim, a longitudinal study of all amputees must be performed.

When compared with previously published work [8] of the experience in Iraq and the early Afghanistan years, our data show that casualties from Afghanistan to have a greater number and a higher level of amputation. This has been alluded to in earlier research [10], but our findings represent a complete cohort of casualties from the entire date range. The heterogeneous nature of warfare results in the variation of statistics seen. Obvious confounding factors to our data include change in standard operating procedures (militarily and medically), change in tactics by the insurgents, equipment improvements, and vehicle factors. The resulting medical and rehabilitation legacy is clear with 265 amputees requiring long-term medical care. We have attempted to place a figure on the financial burden of care for our cohort.

Using published data and an economic algorithm for healthcare costs, we have estimated that over 40 years, £288 million (USD 444 million) will be required to care for our veterans. This is less, per amputee, than calculated for US veterans [4] but more than the cost calculated for civilian trauma amputees investigated in the LEAP study. Historical data collected in 1965 at the Dundee Limb Fitting Centre for 98 veterans of the two world wars and Korean operations revealed on average £9952 (USD 15,350) (data collected in 1965 and published 1999) per limbless servicemen over their lifetime [18]. If we were to extrapolate individual figures for this historical cohort, then the comparative cost, normalized for inflation, is £0.17 million (USD 0.26 million) for life, the lifetime cost of a single amputee. This is similar to values we obtained of £0.16 million (USD 0.025 million) for a tibial component and £0.45 million (USD 0.69 million) for a femoral prosthesis.

The latter years of the conflict in Afghanistan resulted in high numbers of casualties with multiple and complex injuries. Our findings suggest that a long-term facility to budget for our veterans’ prosthetic needs is necessary. Ongoing evaluation and assessment of our injured soldiers will be required to assess the level and specialization of care required as the population ages. It is likely that the described cohort will be subjected to chronic health problems experienced by the general population as well as specific issues as a result of their injuries. These results could be modified if we can develop more effective and sustained medical and social support postmilitary discharge, which would encourage healthier lifestyles and help people develop their skills and earning capacity. This is only possible through long-term financial commitment to health care, social services, and resources such as a single point of care. The British military is embarking on a 20-year study, called the Armed Service Trauma Rehabilitation Outcome study, looking at the health and well-being of amputees and veterans in a case-control cohort study that should hopefully help answer these questions in the future.
